# Coculture model of blood–brain barrier on electrospun nanofibers

**DOI:** 10.3906/biy-1908-42

**Published:** 2020-08-19

**Authors:** Mustafa Görkem ÖZYURT, Ece BAYIR, Şule DOĞAN, Şükrü ÖZTÜRK, Aylin ŞENDEMİR

**Affiliations:** 1 School of Medicine, Koç University, İstanbul Turkey; 2 Graduate School of Sciences and Engineering, Koç University, İstanbul Turkey; 3 Central Research Testing and Analysis Laboratory Research and Application Center, Ege University, İzmir Turkey; 4 Department of Polymer Science and Technology, İstanbul Technical University, İstanbul Turkey; 5 Department of Basic Pharmaceutical Sciences, Faculty of Pharmacy, Hacettepe University, Ankara Turkey; 6 Bioengineering Department, Faculty of Engineering, Ege University, İzmir Turkey; 7 Department of Biomedical Technologies, Graduate School of Natural and Applied Sciences, Ege University, İzmir Turkey

**Keywords:** Blood–brain barrier, electrospinning, methotrexate, 3-dimensional cell culture, transmembrane resistance

## Abstract

The blood–brain barrier (BBB) is a control mechanism that limits the diffusion of many substances to the central nervous system (CNS). In this study, we designed an in-vitro 3-dimensional BBB system to obtain a fast and reliable model to mimic drug delivery characteristics of the CNS. A support membrane of polycaprolactone nanofiber surfaces was prepared using electrospinning. After confirming the fiber morphology and size, endothelial cells (HUVEC) and glial cells were cultured on either side of this membrane. The model’s similarity to in vivo physiology was tested with a home-designed transmembrane resistance (TR) device, with positive and negative control molecules. Finally, 2 doses of methotrexate (MTX), a chemotherapy agent, were applied to the model, and its permeability through the model was determined indirectly by a vitality test on the MCF-7 cell line. Nicotine, the positive control, completed its penetration through the model almost instantly, while albumin, the negative control, was blocked significantly even after 2 days. MTX reached a deadly threshold 24 h after application. The TR value of the model was promising, being around 260 ohm.cm^2^. The provided model proposes a disposable and reliable tool for investigating drug permeability through the BBB and has the potential to reduce the number of animal experiments.

## 1. Introduction

The regulation of the nutrient balance in the human body is strictly regulated, especially in the central nervous system (CNS). Effective maintenance of the CNS is mediated by a specific barrier, called the blood–brain barrier (BBB), which restricts the permeability of some molecules, as well as nutrients from the blood, to the CNS. Generally, lipophilic and smaller molecules can pass through BBB, unlike larger and lipid insoluble ones (Pardridge, 2007). This particularity presents metabolical and physiological maintenance due to selective transport, which provides an optimum medium for neurons in the CNS (Huber et al., 2001).

On the other hand, the selectivity of the BBB may be disadvantageous in clinics. The BBB constitutes a barrier for a number of therapeutic interventions. Many drugs have restricted penetration through the BBB, presenting difficulty in reaching an effective concentration in the CNS for the treatment of several neurological diseases (Deli et al., 2005; Neuwelt et al., 2008). Hence, consideration of the BBB as an obstacle to be overcome is crucial for providing sufficient permeability and bioavailability of a pharmaceutical in clinics.

The BBB includes several cellular elements, i.e. endothelial cells, astrocytes, pericytes, and microglia (Correale and Villa, 2009). Altogether, this cellular structure exhibits unique characteristics and constitutes a highly selective barrier. Specifically, the endothelial cells in the BBB can produce complex and distinct tight junctions compared to those in other tissues (Huber et al., 2001) to regulate diffusivity, along with other cellular elements. Transendothelial electrical resistance (TEER) is a valuable parameter to show BBB effectiveness, as TEER in CNS vasculature is higher than that in other parts of the body (Patabendige et al., 2013; Wilhelm and Krizbai, 2014). Even though both pericytes and astrocytes have major involvement in the BBB, the dominant mechanism is thought to be tight junctions of endothelial cells (Burkhart et al., 2015).

To test the accuracy of drug delivery to the CNS, reproducibility is essential to reliably investigate the effects of different conditions in identical systems. Therefore, many useful in vitro models have been designed (Zhang et al., 2011; Bischel et al., 2016; Qi et al., 2018). The most realistic in vitro models are the ones that mimic the in vivo anatomical conditions properly by including almost all cellular elements or their equivalents (Wilhelm and Krizbai, 2014). These models provide a controlled environment for investigating the effects of treatment on the BBB in a reproducible manner, and are useful for investigating the penetration mechanism of novel therapeutics to the CNS (Abbruscato et al., 2002).

In this study, we designed an in vitro BBB model using an electrospun nanofibrous sheet mimicking the basement membrane of CNS capillaries and a coculture of 2 cell lines, human umbilical vein endothelial cells (HUVEC) and C6 glial cells, to imitate brain endothelial cells and astrocytes, respectively. We, therefore, hypothesize that such a model would mimic the natural metabolic BBB, where transport of certain molecules across the model is blocked or attenuated. The similarity of the model to in vivo BBB and coculture functionality was investigated by measuring transmembrane resistance (TR) and investigating the diffusion of 2 model molecules, nicotine and bovine serum albumin (BSA), through the coculture system. Finally, the permeability of a common chemotherapy drug, methotrexate (MTX), was tested by analyzing its effect on the cell vitality of the human breast cancer cell line MCF-7.

## 2. Experimental procedure

### 2.1. Cell culture, test molecule applications, and reagents

C6 glial cells (gift from Ege University Hospital, Prof. Dr. Gülperi Öktem) and HUVECs (from Ege University Bioengineering Department, Biomaterials and 3D Biointerphases Laboratory stocks) were cultured in DMEM (Thermo Fisher Scientific Inc., Waltham, MA, USA) consisting of 4.5 g/L glucose + 10% fetal bovine serum (FBS) (Biochrome-AG, Berlin, Germany) + 0.1% gentamicin (50 mg/mL) (Thermo Fisher Scientific Inc.). For MCF-7 cells, DMEM/F12 consisting of 4.5 g/L glucose + 10% FBS + 0.1% gentamicin was used. The media were changed 2 times a week in a Class II biosafety cabinet (Esco Technologies, Inc., Horsham, PA, USA). All cell types were kept in an incubator (Thermo Fisher Scientific Inc.) at 37 °C and 5% CO2. Before subculturing the C6 glial (1:3) and MCF-7 (1:3) cells, they were detached using 0.25 wt% Trypsin 0.53 mM EDTA (HyClone Laboratories Inc., Logan, UT, USA), while HUVECs (1:2) were scraped to minimize disruption of cellular tight-junction proteins.

12 wt% polycaprolactone (PCL) solution was used for electrospinning, as it had already been optimized in a previous study in our lab (Zeybek et al., 2014). Next, 10 mL N-N dimethylformamide (Sigma-Aldrich Corp., St. Louis, MO, USA), 10 mL dichloromethane (Sigma-Aldrich Corp.), and 2.4 grams of polycaprolactone (Sigma-Aldrich Corp.) were mixed using MSH-20A magnetic stirrer (WiseStir Limited, Bradford, UK).

Nicotine (positive control) permeability was determined by taking advantage of the color change in the chemical reaction of nicotine (from university stock) and potassium permanganate (KMnO4) (from university stock). This analysis had already been optimized in the Ege University Bioengineering Department, and the most efficient reaction was found with a mixture of 200 μL nicotine solution, 75 μL of KMnO4 (0.0125 M), and 150 μL of NaOH (6.25 M), at 95 °C and 7 min of reaction time. The working concentration of nicotine was decided based on the work of Lockman et al. (2005) as 4.5 μg / mL. Nicotine solution with this concentration was loaded on the inside of the insert. Three samples of 200 μL medium from both sides of the inserts were taken after 2 and 48 h separately and mixed with 75 μL of KMnO4 and then with 150 μL of NaOH (Sigma-Aldrich Corp.). Before the spectrophotometric analysis, mixtures were heated to 95 °C in a plate heater for 7 min for the optimum reaction. The purple color of KMnO4 turned greenish. The samples were read with a spectrophotometer (Spectramax190; Molecular Devices, San Jose, CA, USA) at 610 nm; the amount of nicotine in terms of absorbance in either side of the insert was compared.

Total BSA permeability was measured using the BCA assay. BSA, a 60–70 kDa molecular weight protein that cannot pass through the BBB, was used as a negative control. We loaded 1.83 mg/mL BSA in distilled water inside the inserts and took samples from each side of the inserts 24 and 48 h after application. The samples were diluted according to the BCA assay kit’s (Sigma-Aldrich Corp.) instructions. Five 0.75-μL samples from the medium, with 3 repeats, were taken from each side of the inserts. The samples were diluted by adding 149.25 μL distilled water. For each 150 μL of diluted sample, 150 μL of reaction mixture (73.5 μL reagent A, 73.5 μL Reagent B, 3 μL copper solution) was added; and the mixture was kept in a dark incubator (37 °C) for 2 h. The mixtures were then analyzed in a spectrophotometer (Spectramax190, Molecular Devices Corp., Sunnyvale, CA,USA) at 562 nm.

A common chemotherapeutic agent, MTX (a kind gift from Ege University Hospital, Prof. Dr. Ayfer Haydaroğlu; 0.25%), was applied in 2 doses, 1 µg/0.5 mL and 5 µg/0.5 mL, directly inside the inserts after diluting in DMEM/F12, and compared with the control where no drug had been added.

### 2.2. PCL electrospinning 

The PCL solution was drawn into a 21G-needle syringe and placed in a homemade electrospinning setup consisting of an NE-1000 slow rate pump (New Era Pump Systems Inc., Farmingdale, NY, USA) and a voltage source (Inovenso Inc., Woburn, MA, USA). An aluminum-foil–covered tray was placed perpendicular to the syringe with 15 cm distance. The voltage of 15.7 kV was applied between the tip of the syringe and the tray, while the pump extruded the PCL solution with a working flow rate of 1 mL/h.

### 2.3. Insert design

Once the electrospinning process had finished, electrospun PCL fibers were collected from the aluminum foil and cut into 2-cm diameter circles. These fiber circles were attached to cylindrical inserts (Greiner, catalog no: 665640, Greiner Bio-One GmbH, Kremsmünster, Austria), after removal of their own PET membrane, using silicone O-rings. Active seeding diameter was 0.6 cm (around 1.1 cm2 surface area). Scaffolds were sterilized using ethylene oxide gas for 3 h and aerated for 12 h. The inserts with electrospun PCL membranes attached were placed into 12-well plates with sterile forceps and the inserts were coated with 2 wt% Type B gelatin (Sigma-Aldrich Corp.) solution to improve cell attachment.

To mimic the BBB, we seeded cells on either side of the insert on PCL nanofiber membranes. HUVECs were seeded onto the inside part of the membrane, which represented the blood (luminal) side, while C6 glial cells were seeded onto the outside part of the membrane, which represented the brain (abluminal) side. Finally, MCF-7 cells were seeded on the bottom surface of the 12-well plate, and TR was measured.

### 2.4. Characterization of PCL scaffolds

To determine the mechanical properties of PCL scaffolds, a uniaxial tensile test (DMA, Q800, TA Instruments, New Castle, DE, USA) was performed in tension mode. PCL scaffolds were cut to 5 × 5 × 0.07 mm (length × width × thickness) and mounted between tension clamps on the device. Tests were performed at 0.1 N/min ramp force to 18 N and 23 °C (N = 3) (Lobo et al., 2018). Young’s modulus values were calculated by the slope of the stress–strain curve in the linear region.

The PCL and the gelatin-coated PCL nanofibers were characterized with an FT-IR spectrometer (Spectrum Two FT-IR Spectrometer, PerkinElmer Corp., Waltham, MA, USA). The spectrum of the scaffolds was recorded in the spectral range, 4000–600 cm^-1^, at a resolution of 0.5 cm^-1^. The PCL scaffolds were also characterized by scanning electron microscopy (SEM) in order to observe nanofiber structure.

### 2.5. Observation of cell morphology

To observe the morphology of both C6 and HUVECs with SEM, cells were prefixed with glutaraldehyde (2.5%) in 0.1 M sodium cacodylate buffer for 30 min at 4 °C after washing with isotonic salt solution (pH 7.4). After a treatment of 0.1 M sucrose in sodium cacodylate buffer for 30 min, cells were postfixed with 1.0% OsO_4_ in 1.0 M sodium cacodylate buffer for 30 min. The cells were dehydrated in an ethanol series (35%, 50%, 70%, 85%, 96%, and 100%) after washing 3× in ddH_2_O for 5 min. The cells were held in a hexamethyldisilazane (HDMS, Sigma-Aldrich Corp.) solution for the chemical drying process for 5 min and kept in the fume hood until completely dry. The specimens were sputter-coated with approximately 6 nm of Au before the SEM observation.

### 2.6. Transmembrane resistance measurement

TR was measured using an LCR Meter (GW Instek, Taipei, Taiwan) with a 12-Hz rate through the inside and outside of the insert. The resistances were calculated by multiplying the resistance value with the surface area of the membranes (1.1 cm^2^) (Zhang et al., 2011). TR values were then normalized by subtracting the medium resistance from the obtained values.

### 2.7. Cellular viability test after MTX application

To test the permeability of our model to MTX, we indirectly measured the viability of MCF-7 cells outside the inserts by the MTT assay. 5 mg/mL MTT (Sigma-Aldrich) main stock was diluted 10-fold in serum-free medium. The color change was measured after 3 h of incubation in the dark in an incubator at 37 °C and 5% CO_2_. Formazan crystals were dissolved in dimethyl sulfoxide (Sigma-Aldrich Corp.) by adding 1 mL to each well and shaken for 5 min at room temperature in the dark. The absorbance was measured at 570 nm and 690 nm. The difference between the wavelengths was calculated to report net absorbance as the sign of cell viability.

### 2.8. Statistical analysis

One-way ANOVA was used to test the different conditions in TR measurements, and penetration of control molecules in various time points. MTX groups, on the other hand, were compared using two-way ANOVA between 2, 24, and 48 h of application and 2 doses of drugs along with controls. Multiple comparisons were made using Tukey’s test for one-way ANOVA and the Bonferroni test for two-way ANOVA. Prism 7 (GraphPad, GraphPad Software Inc., San Diego, CA, USA) was used for statistical analyses. The level of significance was selected as P < 0.05.

## 3. Results

After confirming the size and shape of the PCL nanofibers, we attached the membranes onto cell culture inserts and seeded the cells on either side of the membranes. To confirm the accuracy of the model, we measured TR between the inside and outside of the model. Further, we tested the permeability of positive (nicotine) and negative (BSA) controls to reveal the diffusion characteristics of the model. As the final step, we applied MTX and investigated its toxic effect on the cells and permeability through the model.

### 3.1. Glial cells and HUVECs were seeded on both sides of the static model of BBB 

**Figure 1:**
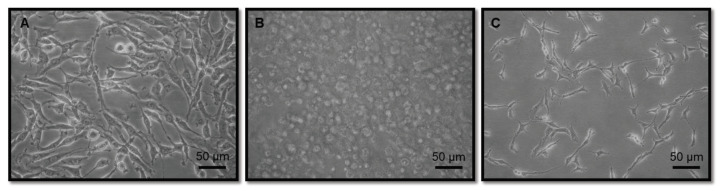
Cellular morphology with 20x magnification is presented on tissue culture plate. A) Glial cells B) HUVEC C) MCF-7. Scale bar is 50 μm.

C6 glial cells were seeded on the outside surface of the insert in a suspension of 200 μL DMEM (4.5 g/L glucose, 10% FBS, 0.1% gentamicin) with a concentration of 4 × 104 cells/insert and incubated for a day. After 24 h of C6 glial cell culturing on the outside surface, HUVECs were seeded on the inside surface of the insert in 700 μL DMEM (4.5 g/L glucose, 10% FBS, 0.1% gentamicin) with a concentration of 1 × 10^5^ HUVECs/insert and incubated for a day. MCF-7 cells were then seeded on the surface of the 12-well plates in 1 mL DMEM (4.5 g/L glucose, 10% FBS, 0.1% gentamicin) with a concentration of 1 × 10^4^ cells per well. Cellular morphology just after passage is shown in Figures 1A–1C.

**Figure 2:**
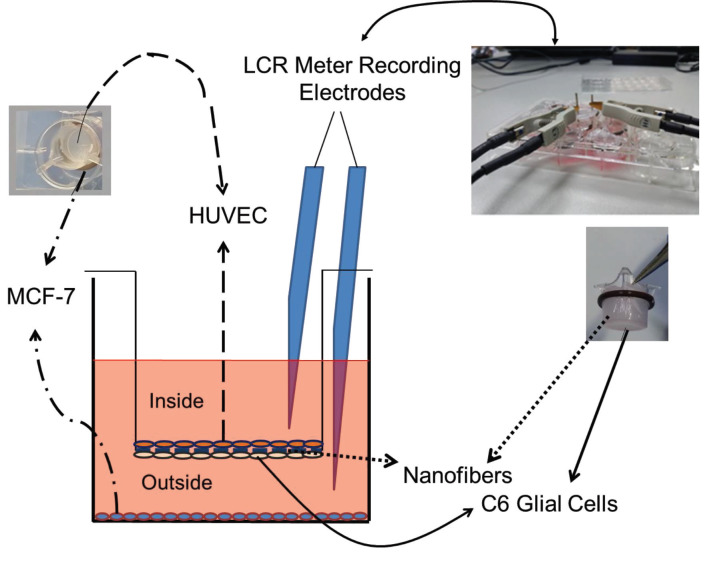
The model insert and TR measurements are illustrated. Using LCR meter, TR was measured between “inside” and “outside” of the insert.

A polystyrene plate was bored with preheated silver rods as electrodes. These electrodes were then glued to the polystyrene plate using cyanoacrylate. One electrode was placed inside the medium within the insert; the other electrode was placed outside, between the insert and the well plate (Figure 2).

### 3.2. PCL scaffold was suitable to retain cellular adhesion

**Figure 3:**
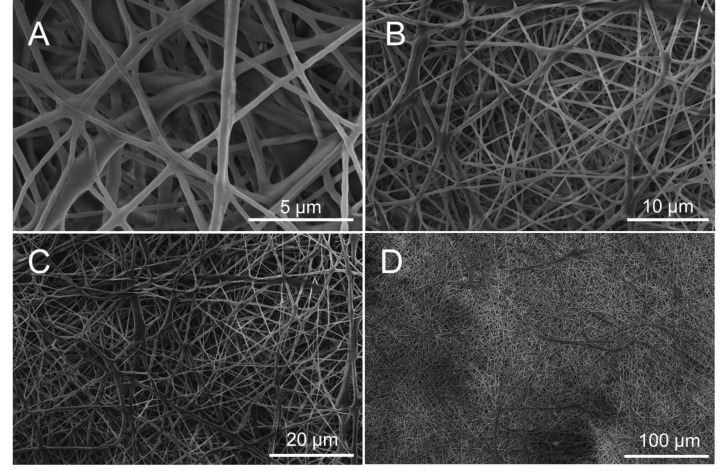
SEM image of electrospun PCL fibers with A) 25000 ×, B) 10000 ×, C) 5000 × and D) 1000 × magnification, without cells seeded. Scale bars are shown on the figures.

Electrospun PCL nanofiber morphology was visualized using SEM. Fiber diameter was between 194.5 and 840.3 nm (339.7 ± 144.4 nm) (Figures 3A–3D). The fibrous sheet thickness of each membrane was less than 100 μm.

Young’s modulus values of the PCL scaffolds were calculated by the slope of stress–strain curve in the linear region. Stress and strain values at break were 2.128 (±0.439) and 267.73 (±17.207), respectively. Young’s modulus value with standard deviation (SD) of 3 PLC scaffolds was 0.609 (±0.056) MPa.

**Figure 4:**
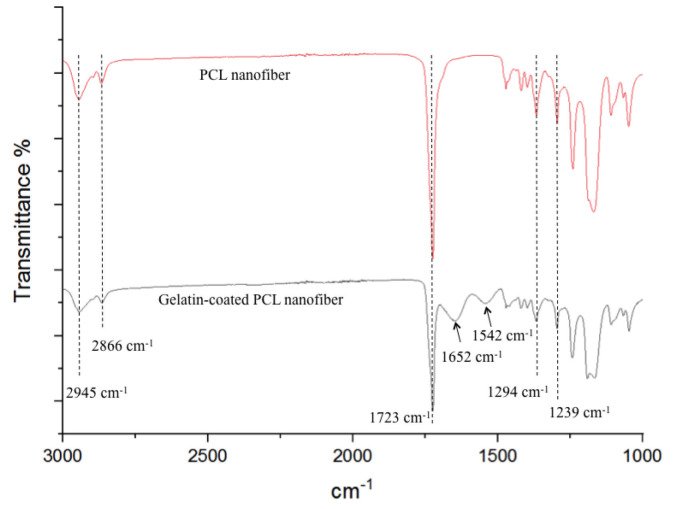
FT-IR spectra of electrospun PCL and gelatin-coated PCL nanofibers between 3000 cm^-1^ and 1000 cm^-1^, at a resolution of 0.5 cm^-1^.

The FT-IR spectra of PCL nanofibers and dried gelatin-coated PCL nanofibers are shown in Figure 4. The characteristic functional group bands of PCL at 2945 cm^-1^ (asymmetric CH_2_ stretching), 2866 cm^-1^ (symmetric CH2 stretching), 1723 cm−1 (ester carbonyl stretching), 1294 cm^-1^ (vibration of C=O and C–C stretching), and 1239 cm^-1^ (asymmetric C–O–C stretching) were observed on both FT-IR spectra (Kuppan et al., 2013; Jia et al., 2016). On the gelatin-coated PCL nanofiber’s spectrum, the characteristic protein bands were present, which are attributed to amide I at 1652 cm^-1^ and amide II at 1542 cm^-1^, along with characteristic bands due to the PCL (Kuppan et al., 2013; Pazhanimala et al., 2019). Moreover, previous characterization of the PCL membrane using the same production parameters in the lab showed around 136° water contact angle (Zeybek et al., 2014).

### 3.3. Glial cells and HUVECs represented normal cellular morphology and confluency on PCL nanofibers

**Figure 5:**
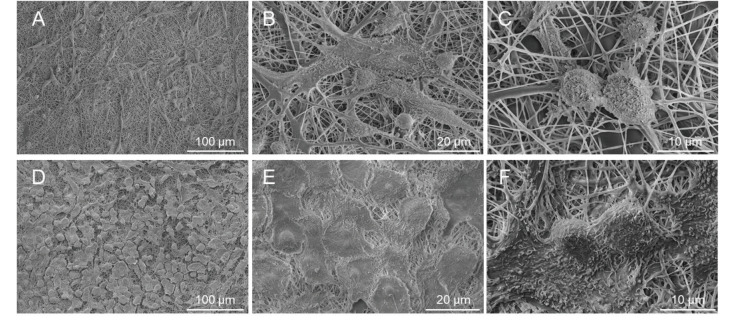
SEM images of the abluminal which is “outside” (A - C) and luminal which is “inside” (D - F) of the gelatin-coated PCL scaffolds. C6 cells on the outside (abluminal - brain) of the insert show heterogeneous protoplasmic astrocyte morphology with many fine processes A) 1000 ×, B) 5000 × and C) 10000 × magnifications. HUVECs on the inside (luminal - blood) of the insert are more confluent than C6 cells; and they show characteristic endothelial cobblestone morphology with close contact with each other D) 1000 ×, E) 5000 × and F) 10000 × magnifications.

SEM images of the C6 seeded on the abluminal side and HUVECs seeded on the luminal side of the gelatin-coated PCL scaffold are given in Figure 5. At low magnification, it can be clearly seen that HUVECs are more confluent than C6 cells, as expected (Figures 5A and 5D). At high magnification of SEM images, it was observed that the C6 cells had heterogeneous protoplasmic astrocyte morphology with many fine processes (Figures 5A–5C); HUVECs had characteristic endothelial cobblestone morphology and were in close contact with each other (Figures 5D–5F).

### 3.4. Transmembrane resistance was the highest when both cells were seeded

**Figure 6:**
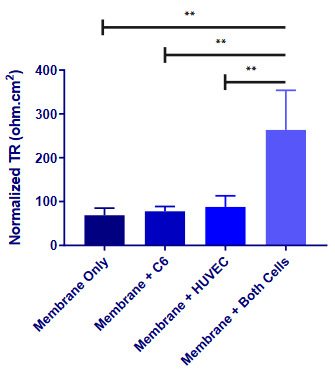
Normalized TR values for each condition. All the resistances were measured using an LCR meter. Readings were multiplied by the surface area of the membranes to standardize the resistance values. Normalization includes subtraction of the electrical resistance value of the cell culture medium from the TR values of each condition. Error bars are the standard deviation. ** P < 0.01. Total of 24 measurements were performed.

We compared TR between several conditions: i) PCL membrane; ii) HUVEC-seeded membrane; iii) glial cell-seeded membrane; and iv) membrane with both cells, separately (Figure 6). No significant differences were found between the membrane-only condition or HUVEC- or glial cell-seeded membrane condition; all were around 60–90 ohm.cm^2^ (P > 0.05). However, both cell-seeded membranes had a TR of 263.53 ohm.cm^2^, which was significantly higher than all other conditions ( membrane only P = 0.0034, membrane + C6 P = 0.0052, membrane + HUVEC P = 0.0083).

### 3.5. Nicotine penetrated the model whereas BSA was effectively arrested 

**Figure 7:**
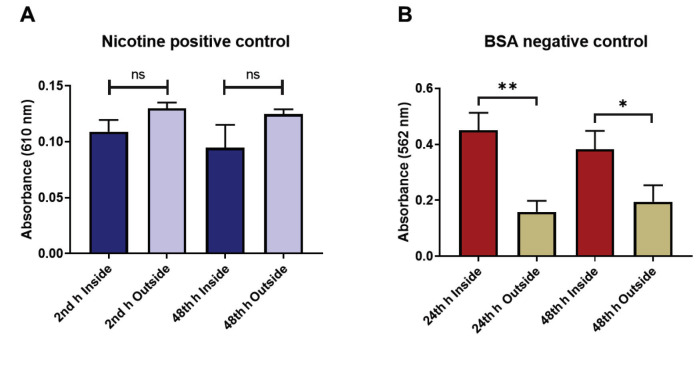
Penetration properties of control molecules that applied inside the model. A) For the positive control, nicotine was introduced inside the insert and samples were taken from both sides of the insert after 2 and 48 h of application. The absorbance of the nicotine is shown in the figure. There is no significant difference between the nicotine concentration inside and outside the insert. B) As the negative control, BSA was used in the same way as in the nicotine application. Samples were taken after 24 and 48 h of BSA application from both sides of the insert and its absorbance were measured. BSA diffusion through the BBB model was significantly hindered on both time points. Error bars are the standard deviation. ^ns^ P > 0.05, * P < 0.05, ** P < 0.01, N = 12 for both nicotine and BSA.

To test the selective permeability of our model, 2 molecules, nicotine and BSA, were loaded inside the inserts having HUVEC and C6 glial cells seeded on the membrane. The penetration of the molecules was investigated using spectrophotometric analysis (Figure 7). Nicotine solution of 4.5 μg/mL was applied. It was found that nicotine passed through the model and reached a plateau 2 h after application (Figure 7A). No notable change was found between the inside and outside of the insert after 48 h (P > 0.05).

For the negative control, 1.83 mg/mL BSA was added inside the insert; the amount of the protein inside and outside of the inserts was detected using the bicinchoninic acid (BCA) assay. After 24 h of application, 75% of the BSA was significantly hindered by the model (P = 0.0012). In addition, almost two-thirds of the BSA was significantly kept inside the insert even 48 h after application (P = 0.0168). It is also noted that the penetrated BSA reached a plateau 24 h after application, as no significant change was observed inside (P > 0.05) or outside of the insert (P > 0.05) after this time point (Figure 7B). The BSA inside was higher than outside at both time points.

### 3.6. High dose methotrexate was more lethal than lower dose after 24 h

**Figure 8:**
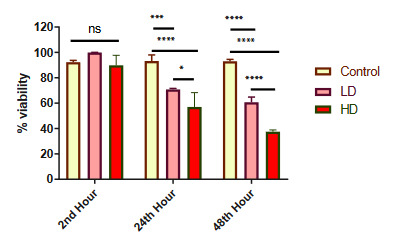
MCF-7 vitality after MTX application to the model. Along with control where no drug has been added, two different doses of MTX, i.e. LD of 1 μg / 0.5 mL MTX, and HD of 5 μg / 0.5 mL MTX were applied inside the inserts. After 2, 24 and 48 h, the vitality of MCF-7 cells was tested using MTT analysis. Net absorbance after drug application was normalized to highest viability value. Error bars are the standard deviation. ^ns^ P > 0.05, * P < 0.05, *** P < 0.001, **** P < 0.0001, N = 12.

The lethal effect of MTX on cell viability, using 3-(4, 5–dimethylthiazol–2-yl)-2, 5-diphenyltetrazolium bromide (MTT) assay, was investigated for 2 concentrations 24 and 48 h after application. We measured the vitality of the MCF-7 cell line after application of the low dose (LD) (1 µg/0.5 mL concentration of MTX) and the high dose (HD) (5 µg/0.5 mL concentration of MTX) inside the inserts (Figure 8).

Although we observed similar viability of MCF-7 2 h after application of both doses, with control having no drug added (all P > 0.05), we found that the HD drug application was significantly more lethal than the LD after 24 h (P = 0.0106) and resulted in less viability than the control to which no drug was added (P < 0.0001). Moreover, the viability of MCF-7 after HD application was observed to be reduced even more 48 h after application compared to LD and control (P < 0.0001). Although similar viability was observed 2 h after LD drug application compared to control (P = 0.1927), there was significant reduction in cellular survival after both 24 h (P = 0.0001) and 48 h (P < 0.0001).

Both LD and HD (P < 0.0001) application significantly reduced cellular viability timewise between 2, 24, and 48 h.

## 4. Discussion

Drug delivery methods have recently been optimized to target the CNS; however, the BBB is a significant obstacle, as it restricts the diffusivity of the active molecule(s) from the blood. To reach a therapeutic concentration in the CNS, a molecule must pass this physiological barrier. Therefore, we designed a 3-dimensional (3D) experimental model of the BBB and tested its efficacy using control molecules, measuring TR of the model and lethal effect of a common chemotherapy drug. All of these trials were performed in different samples to prevent additional effects, such as nicotine’s alteration of the BBB’s permeability (Lockman et al., 2005). Here, we discuss these original findings from this study: the effect of cell selection, measurement, and significance of the positive and negative controls, obtained TR values, and MTX diffusivity to determine the overall efficacy of the model.

### 4.1. Cellular composition of the proposed model

The components of the model were the cell lines used to produce the 3D structure, along with electrospun PCL nanofibers. An accurate selection of the cells is indispensable for an appropriate model to mimic the properties of the BBB in vivo. Endothelial cells of the brain create a unique cellular structure of tight junctions that cause high selectivity (Joó, 1996). Although specific endothelial cell lines or primary cultures derived from the brain capillaries are the best choices, HUVECs have also been shown to be a good option due to their growth kinetics (Kalashnik et al., 2000), and have been used in various BBB models as a reliable cellular component (Yeon et al., 2012). HUVECs are easy to cultivate, proliferate fast, and create cellular junctions similar to tight junctions of in vivo endothelial cells (Pawlowski NA, 1988). In addition, it has been shown that nonneuronal endothelial cells, such as HUVEC in our study, are capable of being induced to have various blood–brain barrier properties via astrocytes (Akiyama et al., 2000). In the aspect of astrocytes, C6 glial cells have also been used for BBB studies (Raub, 1996); they were selected as they proliferate fast, improving the reproducibility of the model, and produce extracellular matrix (Malek-Hedayat and Rome, 1992), providing potential to increase the molecular selectivity. On the other hand, MCF-7 cells do not belong to the CNS; however, clinically, symptomatic metastasis to the CNS can be observed in patients with metastatic breast cancer (Weil et al., 2005). MCF-7 was selected to represent a tumor tissue since their properties are well defined in the literature, including their growth kinetics as well as the existence of receptors like estrogen and progesterone receptors (Brooks et al., 1973).

### 4.2. Nicotine and BSA permeability of the model

Nicotine was used as a positive control due to its widely known ability to pass the BBB (Lockman et al., 2005). Animal behavioral studies that have investigated the brain uptake rate of nicotine have revealed that exposure of 4.5 mg nicotine solution/day resulted in consumption of 4.5 μg/mL nicotine hourly (Lockman et al., 2005). We applied similar doses of nicotine to our in vitro model and found that it fully penetrated through the BBB and reached equilibrium in only 2 h.

Due to its high molecular weight, BSA was used to detect the blocking capacity of the model. It was shown that the permeability of BSA is limited through BBB in both in vivo (Shimon-Hophy et al., 1991) and in vitro (Smith and Borchardt, 1989) models. We used the serum-free medium to increase the precision of BCA analysis, as BSA in the serum might affect the findings. However, cell-origin proteins, cellular consumption of BSA, and heat-induced degradation of BSA might affect the amount of protein detected, even though heat degradation of BSA is unlikely at 37 °C (Borzova et al., 2016). In order to minimize those effects, we analyzed all of the inserts in the same environmental conditions and then compared the total amount of proteins inside and outside of the inserts, and assumed that the majority of the detected protein was BSA. We found significant blocking of the protein, as almost 75% of the molecule was kept inside the insert even after one day of BSA application. Similarly, almost 65% of the BSA was still located inside the insert at the end of the second day, even though the total amount of protein was lower compared to the first day, probably due to cellular consumption.

### 4.3. MTX analysis to test drug retention of the model

To investigate the accuracy of the model, MTX was applied to further prove the blocking properties. The doses applied were 5 µg/0.5 mL and 1 µg/0.5 mL. We chose these concentrations according to the studies that involve intravenous and intraventricular injections in vivo (Shapiro et al., 1975). MTT analysis showed that our model could not block the MTX penetration for sustained exposure if it lasted more than 24 h. The reduction in cell viability outside the insert was lower as time passed, which shows remarkable penetration. This could be because MTX might first disrupt the model’s cellular elements since it is highly toxic, especially for HUVECs, as they are the first cells encountering the MTX. Eventually, MTX reaches the bottom of the plate, where MCF-7 cells are located.

When most of the drugs are applied intravenously, a relatively lower concentration reaches the brain; the BBB is the main mechanism that limits the effective transfer of the drug to the CNS (Mangas-Sanjuan et al., 2010). Here, we investigated the model’s accuracy according to the effective concentration reached around the brain (outside of the insert) and demonstrated that MTX disrupts the cellular component of the model while passing through the BBB (inside of the insert). However, MTX conjugated nanoparticles increase the efficacy of targeting and reduces the collateral damage to the healthy cells (Kohler et al., 2005). This system might be applied to our model and further proves that the reduction in cellular death in the BBB model can improve the MTX retention inside the inserts to show the reliability of this 3D coculture system.

### 4.4. Comparison of the model with the currently available systems

We presented an improved 3D coculture model of the BBB to be used in CNS toxicity and drug discovery studies. We tested control substances that were either known to be blocked by or pass through the BBB. Additionally, we investigated if TR obtained from the model was close to TEER values of in vivo and other in vitro BBB models. More importantly, SEM images showed efficient cellular attachment on either side of the scaffold.

The main advantage of the proposed model is swift production of the scaffold material, fast/efficient testing of the new substances due to easy production of the model, and its low cost. Some similar studies use various primary cells, such as neonatal rat glial cells (Gaillard et al., 2001; Abbott et al., 2012). However, these systems need isolation, characterization, and optimization of each cell batch when obtained from animals, and may result in unpreventable contamination of the other cellular elements. This, in turn, may result in some variations in the produced in vitro BBB and demands testing/characterization before each use. Moreover, commercially available inserts, such as polycarbonate cell culture inserts, have uniform pore sizes but are more limited in mimicking in vivo conditions as well as already lower membrane-only TEER than our model used in this study (Eigenmann et al., 2013; Wuest et al., 2013).

The current system is an example of a static model, which does not effectively represent actual dynamic conditions given that in vivo conditions include but are not limited to continuous vascular flow and variabilities in blood pressure. Dynamic BBB models propose more complex systems and propose to mimic the in vivo environment better compared to static models, as the dynamic models include vascular flow as well as related extracellular matrix and cellular components (Neuhaus et al., 2006; Cucullo et al., 2011). In addition, these dynamic systems aim to mimic the actual environment in vivo by providing a microfluidic environment replicating multicellular mechanics and architecture which is not comparable with the static models (Xu et al., 2016). However, what we propose in this study would be a first step in investigating the penetration of drugs and molecules with low cost and in a faster manner than dynamic systems, allowing an increased number of trials in a short time with decent proximity.

Although the control substances had similar penetration kinetics as we hypothesized, the TR value of our model measured by LCR meter was about 30% of the TEER value of the BBB model in Patabendige et al. (2013). The TR value of our model (260 ohm.cm2) was within the TEER values of the other cell culture models that have previously been reported between 200–800 ohm.cm2 (Abbott et al., 2012; Xue et al., 2013; Qi et al., 2018). However, a value of around 1900 ohm.cm2 has been found in vivo in the brain microvascular endothelium (Crone and Olesen, 1982), which is another disadvantage of the proposed model. Despite the lower TR value of the current model compared to the natural BBB, other characteristics of the model represent the physiological conditions of the in vivo BBB. The TR value can be increased by implementing dynamic flow conditions, which alone does not always provide the necessary physiological conditions to mimic the natural BBB (Cucullo et al., 2008; Appelt-Menzel et al., 2017). The current static model, on the other hand, provides instant nicotine penetration, which is compatible with in vivo dynamic conditions (Lockman et al., 2005). Additionally, albumin penetration in vivo was observed to be very slow (Banks et al., 2000), which is sufficiently demonstrated by our model, which kept two-thirds of the BSA inside the insert even after 48 h. Moreover, the in vitro models having resistance values above 150 ohm.cm2 are regarded as sufficient in contemporary pharmaceutical research, that value is well below than the model presented in this study (Appelt-Menzel et al., 2017).

The factors affecting the TR resistance in the BBB models include cellular confluence on either side of the membrane and their cell–cell junctions, porosity and thickness of the nanofibers, types of cells seeded, initial number of cells seeded, temperature, shear stress, and ionic composition of the medium (Srinivasan et al., 2015). Increased cell confluence, higher cellular contact, higher initial seeded cell number, and a thicker membrane could increase the resistance of the model, whereas higher conductance ionic composition of the medium, higher porosity, and selection of the cells (e.g., cells lacking tight junction properties) could result in lower TR. In vivo BBB has optimum blood ionic concentration, cellular and basal lamina structure. These parameters could only be provided by our model up to some extent, and this causes a significant difference of TR between our model and in vivo BBB. Therefore, this model can be improved by further optimization to be used as the first step in clinical drug trials to test BBB permeability.

Another issue of the model to be considered before application is the variability/randomness of the nanofiber diameters that may result in slight deviations in the cellular attachment. Randomly aligned nanofibers and cellular contacts on either side increase the proximity of the proposed model and mimic the natural BBB better. Having appropriate basal membrane is crucial for mimicking physiological homeostasis and allowing cellular contact. Seeded cells on randomly aligned nanofibers tight enough to retain cells allow cellular connections laterally, which may allow tight junction formation on the luminal side and perineuronal nets, as well as neural interstitial matrix on the abluminal side. PCL nanofiber scaffolds with current parameters are shown to support cellular attachment (TR results) “and can accommodate different cell types than used in this study (Zeybek et al., 2014). Changing parameters to produce PCL nanofibers may affect structure and therefore functional outcome. Several parameters, namely source to ground distance and concentration of PCL solution, may change pore size and nanofiber diameters as well as tensile strength. Elastic modulus, for instance, may change from around 4 MPa to levels of several tens of MPa by changing the source to ground distance as well as the rotation of the base if included (Gaumer et al., 2009). Moreover, these parameters also change fiber angle and pore size (Gaumer et al., 2009). Altogether, increased pore sizes may reduce cellular attachment, and uniform fiber production may affect cell proliferation and migration. 

To overcome these disadvantages and in order to produce a more effective and proximate model to in vivo BBB, the compactness of electrospun fibers should be optimized further by using materials with higher compatibility with endothelial and glial cells; e.g., collagen could be used instead of PCL. Electrospun collagen helps cells attach and proliferate easily (Chen et al., 2010), which might further increase the number of tight junctions, as well as the TR value. The initial concentration of seeded cells on the electrospun PCL could be optimized further for the increased confluency of C6 glial cells and HUVECs that contribute to the resistance. We selected HUVECs and C6 glial cells due to their ability to create tight junctions (Pawlowski NA, 1988) and extracellular matrix production (Gladson, 1999), respectively, both of which contribute to molecular selectivity. Finally, a different source of cells, such as primary BBB elements, may be used to optimize the cellular physiology in the model, which could mimic the in vivo BBB in a more accurate manner, but this requires extensive characterization before each use, which is a drawback.

## Acknowledgments

This project was supported by TÜBİTAK (The Scientific and Technological Research Council of Turkey) by the 2209-A program. We would like to thank EBİLTEM (Ege University Science and Technology Application and Research Center), Ege University Bioengineering Department Biomaterials and 3D Biointerphases Laboratories, and colleagues for supporting this project. We also thank Ege University Faculty of Medicine Oncology Department and Prof. Dr. Ayfer Haydaroğlu for providing MTX and Prof. Dr. Gülperi Öktem for providing C6 glial cells. 
